# The Properties of Modified Bagasse Fiber/Nano-TiO_2_ Composite Asphalt in a High-Temperature and High-Humidity Salt Environment

**DOI:** 10.3390/ma16175996

**Published:** 2023-08-31

**Authors:** Zhenxiang Xie, Liansheng Tang, Mengru Tao, Fangjian Yang, Qilin Zhong

**Affiliations:** 1 School of Earth Science and Engineering, Sun Yat-Sen University, Zhuhai 519082, China; xiezhx25@mail2.sysu.edu.cn (Z.X.); taomr@mail2.sysu.edu.cn (M.T.); zhongqlin5@mail2.sysu.edu.cn (Q.Z.); 2 School of Architectural Engineering, Guangzhou Institute of Science and Technology, Guangzhou 510540, China; 3 Guangdong Provincial Key Laboratory of Geodynamics and Geohazards, Zhuhai 519082, China; 4 224 Harbert Center, Department of Civil Engineering, Auburn University, Auburn, AL 36849, USA; fzy0022@auburn.edu

**Keywords:** pavement engineering, tropical coastal area, asphalt binder, modified bagasse fiber, rheological property

## Abstract

The southern tropical coastal areas of China are high-temperature and high-humidity salt environments, which hinder the durability and service life of ordinary asphalt pavement. To enhance the durability of asphalt pavement in these areas, modified bagasse fiber combined with nano-TiO_2_ was used to improve the corrosion resistance of asphalt pavement in high-temperature and high-humidity salt environments. The micro-morphology, high-temperature oil absorption, high-temperature heat resistance, and hygroscopicity of bagasse fiber modified using three silane coupling agents combined with NaOH were compared, and the best silane coupling agent/NaOH modification scheme for bagasse fiber was found. Based on conventional physical tests (penetration, softening point, ductility), rheological property tests (rotational viscosity, dynamic shear rheological test, multi-stress creep recovery test, linear amplitude scanning test), and a four-point bending fatigue test of the asphalt mixture, the properties of modified bagasse fiber asphalt binder and mixture after cyclic dry–wet erosion under pure water and salt solution (NaCl, Na_2_SO_4_) were determined, and the effects of the erosion environment and fiber ratio on the basic physical and rheological properties of the asphalt were clarified. Compared with the silane coupling agents KH550 and KH590, the bagasse fiber modified with KH570/NaOH had a better high-temperature oil absorption capacity, heat stability capacity, and matrix asphalt compatibility. The worst erosion environment was Na_2_SO_4_, but the increase in test temperature and fiber content weakened the sensitivity of the asphalt binder performance in different erosion environments. The erosion capacity order was as follows: Na_2_SO_4_ > NaCl > pure water. In the worst erosion environment, 0.5% modified bagasse fiber/Nano-TiO_2_ asphalt binder (*B_n_*_−570−0.5_) had the best corrosion resistance in a high-temperature and high-humidity salt environment. The penetration, softening point, creep recovery rate *R*_3.2_, non-recoverable creep compliance *J_nr_*_3.2_, and fatigue life after long-term aging (with 5% strain) of *B_n-_*_570-0.5_ were, respectively, increased by −16.9%, 37.5%, 37.95%, −27.86%, and 38.30% compared with unblended base asphalt binder (*B*). In addition, the four-point flexural fatigue life of *B_n-_*_570-0.5_ was 169.2% higher than that of the unblended base mixture.

## 1. Introduction

Tropical coastal areas are often high-temperature and high-humidity salt environments. The asphalt pavement built in these areas needs to overcome problems, such as peeling, looseness, pits, and shifts, that are prone to occurring after long-term operation [1,2]. Asphalt mixture is a viscoelastic material, which is composed of asphalt, mineral powder, and coarse and fine aggregates [3]. An increase in temperature causes the asphalt in the mixture to expand and soften, the interface strength of the aggregate and asphalt to decrease, and the overall stiffness to reduce [4].When the asphalt mixture is exposed to a high-humidity environment for a long time, water enters its structure through gaps or other paths, forcing the asphalt glue film to fall from the aggregate surface due to the adsorption force of water on the aggregate surface being stronger than the adsorption capacity of asphalt on the aggregate surface. The dynamic water pressure generated by the driving load is then bound to the outside of the road surface [5]. The atmosphere in coastal areas contains a lot of soluble salt. The salt enters the pavement structure through rainfall and sea fog, invades the interface between the aggregate and asphalt binder, and acts with water to accelerate the decay of cementation strength in the asphalt–aggregate contact area [6]. Therefore, determining how to reduce the influence of saline erosion on asphalt degradation in coastal areas and improve the service life of pavement is an important research direction in respect to asphalt material improvement.

Nano-TiO_2_ is often used to improve the physical and mechanical properties of asphalt and asphalt mixtures due to its extremely small size and large specific surface area [7]. Shafabakhsh (2014) [8] and Azarhoosh et al. (2016) [9] found that nano-TiO_2_ reduced the sensitivity of the cohesion between modified asphalt and aggregate under high-temperature and water erosion conditions. Daroonparvar et al. (2014) [10] found that nano-TiO_2_ could improve the chloride erosion resistance of the material. Liao et al. (2021) [11] found that nano-TiO_2_ had a good effect on improving the photoaging resistance of SBS-modified asphalt. Tian et al. (2020) [12] selected nano-TiO_2_/CaCO_3_ combined with basalt fiber for compound modification of asphalt and found that a nano-material combined with fiber can have a superposition effect on the improvement in asphalt performance. Moreover, it can simultaneously improve the high-temperature stability and low-temperature crack resistance of asphalt. This verified the effectiveness of the nano-material combined with fiber composite improvement of asphalt.

Fiber is one of the most common materials used to strengthen cement concrete and asphalt mixtures [13]. In general, there are two main types of fibers: synthetic fibers (man-made fibers) and natural fibers (plant fibers). Synthetic fibers commonly used to improve the performance of asphalt mix include polypropylene fiber, carbon fiber, glass fiber, lignin fiber, and basalt fiber [14,15,16,17,18], but these mineral fibers or polymer fibers are relatively expensive and difficult to use in a wide range of applications. In recent years, natural fibers have received more attention in the field of asphalt improvement because of their low cost and environmental benefits. Li et al. (2022) studied the performance of five different plant fibers on asphalt binders, and found that corn stalk fiber had the highest surface free energy, had the strongest adhesion to asphalt binders, and significantly improved the road performance of asphalt binders [19]. Laiana et al. (2020) [20] found that banana fiber can improve the rutting resistance of the SMA mixture and reduce diseases such as asphalt pavement pushing and loosening under high-temperature conditions. Natural bagasse fiber, whose main composition is similar to sisal fiber and corn stalk fiber, is also commonly used to improve the properties of SMA [21] and SBS-modified asphalt [22,23]. Combined with the research results of Mansor et al. (2018) [24], He et al. (2020), and Li et al. (2022) [25,26], found that, after the natural bagasse fiber is chemically modified under certain conditions, its high-temperature oil absorption and heat resistance are improved, the impurities on the surface of the fiber (such as glue, hemicelluloses, etc.) are removed more fully, and the compatibility with asphalt binder is better. These factors are conducive to improving the overall high-temperature stability, water stability, and low-temperature crack resistance of the asphalt material.

In summary, at present, scholars mainly focus on studying the high-temperature and high-humidity resistance of the asphalt binder and mixture improved by different fiber materials or different nano-materials alone, paying little attention to salt corrosion factors in tropical coastal areas and rarely applying nano-materials and fibers in combination, especially the combination of nano-materials and natural fibers. Therefore, the current asphalt improvement methods are not economical and adaptable in tropical coastal environments.

This study was based on the high-temperature and high-humidity salt environment in Zhanjiang, a tropical coastal city in southern China, which has experienced long-term erosion. Zhanjiang also has a well-developed local sugar industry that generates natural bagasse fiber waste [27]. We proposed to conduct this study to improve the resistance of common asphalt to high-temperature, high-humidity salt conditions by combining modified bagasse fiber with nano-TiO_2_. Firstly, the bagasse fibers were modified with NaOH in combination with different silane coupling agents. Changes in the microscopic morphology, high-temperature oil absorption, high-temperature heat resistance, and hygroscopicity of the modified bagasse fibers were observed to determine the best solution by which to improve the modified properties of bagasse. Secondly, based on a self-designed indoor salt erosion dry–wet cycle treatment, asphalt samples with different fiber contents and different erosion environments were subjected to rotational viscosity, a dynamic shear rheometer (DSR), multiple stress creep and recovery (MSCR), and a linear amplitude sweep (LAS), as well as four-point bending fatigue tests, to analyze the effects of high-temperature and high-humidity salt coupling on the performance of modified bagasse fiber/nano-TiO_2_ asphalt binder and mixtures. Finally, we determined the best modified bagasse dosage scheme to resist the worst erosion conditions.

## 2. Materials

### 2.1. Base Asphalt and Mineral Powder

In this study, A-70^#^ base asphalt was used, which was supplied by Maoming Petrochemical. Table 1 shows the measured properties of the asphalt. The selected mineral powder is limestone mineral powder produced in Suixi, Zhanjiang, and the indexes are shown in Table 2. The performance test results of the above materials are in accordance with JTG D50-2017 and JTG F40-2004 requirements [28,29].

### 2.2. Bagasse

The bagasse was from Zhanjiang, China, a city with a well-developed sugar industry and abundant bagasse raw materials. Due to the lack of a specific specification, reference is made to JTT 533-2004 [30] to evaluate the basic properties of bagasse fibers, as shown in Table 3.

### 2.3. Salt Solutions

The salts in the Zhanjiang area comprise mainly chloride and sulfate. A 10% NaCl solution and 10% Na_2_SO_4_ solution were used as the simulated environments for salt erosion in the dry–wet cycle experiment [31,32]. The NaCl used in the experiment is a white tetragonal crystal, slightly deliquescent, soluble in water and glycerol, and insoluble in ethanol and hydrochloric acid [33]; Na_2_SO_4_ is a white crystalline powder, hygroscopic, soluble in water, and insoluble in ethanol [34]. The specific technical parameters are shown in Table 4 and Table 5.

### 2.4. Nano-Material

**Figure 1:**
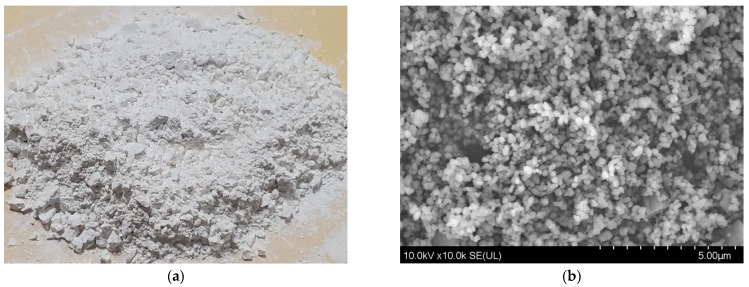
Nano-TiO_2_. (**a**) Image of nano-TiO_2_; (**b**) SEM Image (10 KX).

The basic physical parameters of the selected nano-TiO_2_ modifier are shown in Table 6. Figure 1 shows the daily state and microscopic characterization images of nano-TiO_2_.

### 2.5. Silane Coupling Agent

A silane coupling agent is a class of silane with organic functional groups whose molecules have both reactive groups that can chemically bond with inorganic materials (glass, silica sand, metals) and organic materials (synthetic resins), which can build a “molecular bridge” between the interface of inorganic and organic materials, connecting two materials with different properties, improving the performance of composite materials, and increasing the strength of the bond [35]. As shown in Table 7, three common silane coupling agents, KH550, KH570, and KH590, were used to further modify the bagasse fibers after NaOH treatment. The best NaOH–silane coupling agent scheme among them was identified by observing the physical indices of the modified bagasse fibers.

### 2.6. Sample Preparation

#### 2.6.1. Preparation of Modified Bagasse Fiber

The modified bagasse fibers were prepared as follows: Bagasse fibers of 4–6 cm in length were soaked in NaOH solution of 5% mass fraction at 60 °C for 60 min. We separated the fibers from the NaOH solution and air-dried them in an oven at 40 °C. Silane coupling agent, deionized water, alcohol solution, and acetic acid were mixed at the ratio of 1:1:20 to prepare a silane coupling agent dissolution solution with a pH value of 4. We then stirred the dissolution solution for 40 min using a magnetic stirrer. The air-dried bagasse fibers were soaked in the silane coupling agent dissolution solution for 2 h. After soaking, we washed the bagasse fibers and dried them in an oven at 40 °C.

#### 2.6.2. Preparation of Modified Bagasse Fiber–TiO_2_ Asphalt Binder

**Figure 2:**
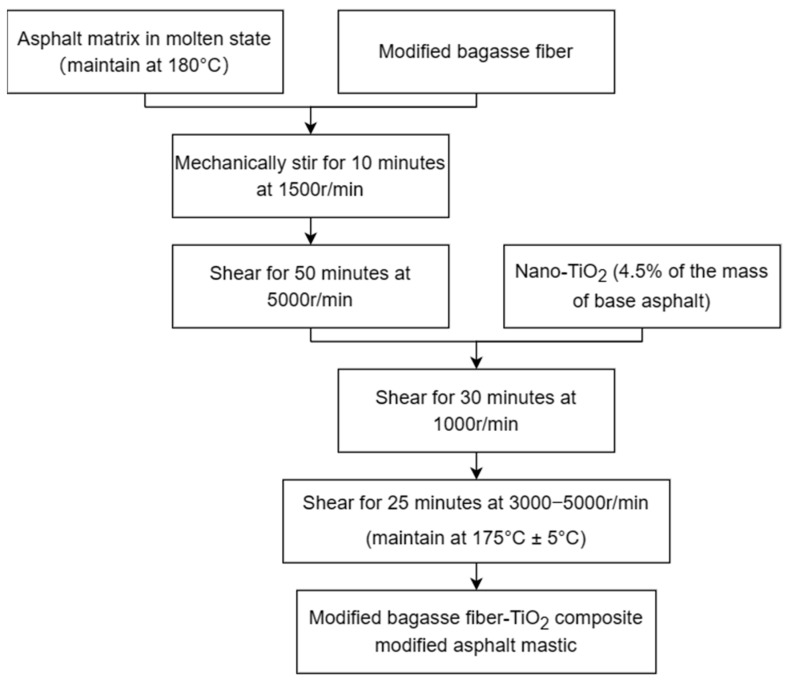
Preparation process of the composite-modified asphalt.

The modified bagasse fiber–TiO_2_ asphalt binder was prepared using a high-speed shear and a constant temperature magnetic heating stirrer. During preparation, the temperature of the asphalt was maintained between 170 °C and 180 °C. The preparation process is presented in Figure 2.

## 3. Experimental Methods

### 3.1. Modified Bagasse Stability Test

Using the net basket method (AASHTOT305-97) for asphalt precipitation leakage testing, we tested the amount of asphalt dripping from the net basket at 130 °C and 160 °C for 3 h (recorded every 30 min) to understand the high-temperature oil absorption performance of the modified bagasse fiber. We kept the fibers at a constant temperature of 210 °C for 0.5 h, 3 h, and 5 h for the heat resistance experiments in order to understand the heat loss rates of different fiber samples. The fiber samples were placed in an airtight permaculture chamber at 35 °C and a relative humidity of 90% for 20 d, and the humidity of the fiber samples was recorded at 2 d, 5 d, 10 d, 15 d, and 20 d to study the resistance of different fiber samples to moisture absorption.

### 3.2. Aging Test

Referring to the experimental scheme of Ferrotti et al. (2019) [36], a powder-to-asphalt ratio of 1:1.2 (mass ratio of mineral powder to asphalt) was used for mastic preparation. Referring to the methods of T0610 and T0630 in JTG E20-2011 [37], the asphalt specimens were aged for a short period of 75 min using a rolling thin-film heating oven (the temperature in the oven was controlled at 163 °C; the rotational speed of the turntable was 15 r/min) and a long period of 20 h using a pressure aging vessel (the temperature in the oven was controlled at 90–110 °C; the air pressure was 2.1 Mpa).

### 3.3. Wetting–Drying Cycle Test under Salt Corrosion Conditions 

**Figure 3:**
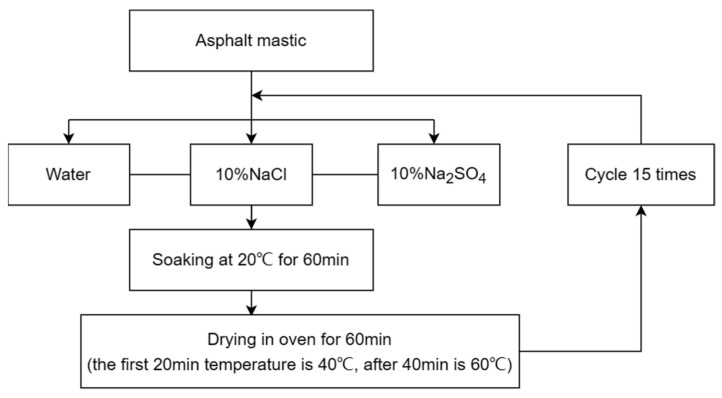
Experimental procedure of dry and wet cycles under salt corrosion conditions.

To simulate the climatic environment of high temperature, high humidity, and high salt in the Zhanjiang area in summer and autumn, indoor experiments in respect to the dry and wet cycles of salt erosion were self-designed. According to the summer and autumn surface temperature range in the Zhanjiang area, the low-temperature environment for setting asphalt mastic samples was 20 °C and the high-temperature environment was 65 °C. The specific cyclic process is shown in Figure 3.

### 3.4. Rotational Viscosity Test

In order to study the resistance of modified bagasse fiber asphalt with different blends to shear deformation flow in a high-temperature and high-humidity salt environment, the rotational viscosities of base asphalt and modified bagasse fiber asphalt with different dosing levels were measured at temperatures of 100 °C, 120 °C, 135 °C, 150 °C, and 165 °C using an RV-type viscometer (speed: 20 r/min) [38] and the corresponding viscosity–temperature curves were obtained.

### 3.5. Dynamic Shear Rheometer Test (JTG E20-2011, T0602)

Dynamic shear rheometer (DSR) testing, using parallel-plate geometry, is currently the most common type of oscillatory testing carried out for rheological characterizations in bituminous materials [39]. The test apparatus in this study was an MCR302 dynamic shear instrument produced by Anton Par Austria. We used the temperature scanning mode, the experimental temperature was 40–70 °C, the interval was 3 °C, the control strain was 1.2%, and the experimental frequency was 10 Hz. 

### 3.6. Multiple Stress Creep and Recovery Test (MSCR)

The test was conducted in two stress stages, with the first stress stage using a control stress of 0.1 kPa and 20 cycles of loading (no data were recorded for the first 10 cycles to condition the specimens and reduce the variability of the results); the second stress stage used a control stress of 3.2 kPa (10 cycles of loading) for a total of 30 cycles. The loading process for each cycle was as follows: loading at constant stress for 1 s and recovery at zero stress for 9 s. There was no time interval between the application of each stress, for a total duration of 300 s. *R*_0.1_ and *R*_3.2_ represent the ratio of recoverable strain to peak strain during loading for 1 s and unloading for 9 s at 0.1 kPa and 3.2 kPa, respectively, which shows the deformation recovery ability of the modified asphalt specimens. *J_nr_* is the ratio of residual strain to applied stress in the asphalt specimens during the unloading process, which reflects the ability of asphalt mastic to resist permanent deformation. The smaller the value of *J_nr_*, the stronger the resistance to high-temperature deformation and the better the high-temperature rutting resistance [40,41].

### 3.7. Linear Amplitude Sweep Test (LAS)

In order to investigate the fatigue resistance of asphalt mastic with different doping levels of modified bagasse fiber for short-term and long-term aging, linear amplitude sweeps were performed on specimens treated in a rolling thin-film oven test (RTFOT) and pressure aging vessel (PAV) to identify the damage characteristics of asphalt samples [42]. A controlled strain loading method was used to increase the amplitude of the test-loaded sine wave load linearly from 0.1% to 30% at 10 Hz (for a total duration of 300 s) to obtain the change in stress magnitude of the modified bagasse fiber asphalt mastic samples at different doping levels under linearly increasing strain conditions. In combination with the VECD model, the fatigue life of the modified bagasse fiber asphalt (after wetting–drying cycle test disposal) was predicted at 2.5% and 5.0% strain conditions.

### 3.8. Four-Point Bending Fatigue Test

To study the fatigue life of modified bagasse fiber asphalt mixtures at different strain levels, fatigue tests were conducted on modified bagasse fiber asphalt mixtures with different doping levels after long-term aging. According to the requirements of the AASHTO T321 standard [43], and with reference to the experiment of Shafabakhsh et al. (2019) [44], strain levels of 400, 700, and 1000 were set as the control conditions, and the fatigue life of modified bagasse fiber asphalt mixes with different dosing levels was recorded when the stiffness was reduced to 50% of the initial stiffness. The loading mode was sinusoidal loading at a temperature of 20 °C and a loading frequency of 10 Hz. The mixtures were treated with a wetting–drying cyclic erosion test prior to the fatigue test.

## 4. Results and Discussion

### 4.1. Effects of NaOH/Silane Coupling Agent on Bagasse Fiber Modification

#### 4.1.1. High-Temperature Oil Absorption

**Figure 4:**
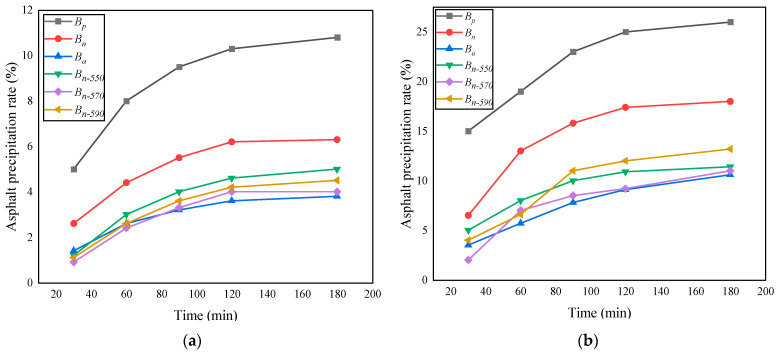
Results of asphalt precipitation tests at different temperatures: (**a**) 130 °C; (**b**) 165 °C.

According to Cui et al. (2022) [45], the thermal stability of bamboo fiber treated with a silane coupling agent is enhanced, and its adhesion is 66.7% higher than that of untreated fiber, which means that modified fiber can adsorb more asphalt to form a gel with high cohesiveness and hinder the development of micro-cracks in asphalt at high temperature more effectively. As can be seen from Figure 4, the precipitation amount of fiber increases the fastest within 90 min of initial heating. The rate inflection point is 120 min, and the asphalt precipitation rate of 120–180 min changes within 10% and tends to be stable. The precipitation rate of *B_p_* (130 °C, 180 min) is 10.8%, which is 71.4%, 184.2%, 116%, 171%, and 140% higher than those of *B_n_*, *B_a_*, *B_n-_*_550_, *B_n-_*_570_, and *B_n-_*_590_, respectively. The precipitation rate of *B_p_* (165 °C, 180 min) is 26.1%, which is 142% of its own value at 130 °C. This is 44%, 145%, 128%, 143%, and 168% higher than the values of *B_n_*, *B_a_*, *B_n-_*_550_, *B_n-_*_570_, and *B_n-_*_590_, respectively. The asphalt modified by *B_a_* has good thermal stability, and the precipitation rate is only 3.8% at 130 °C (180 min) and 10.6% at 165 °C (180 min). The closest parameter to the *B_a_* effect is *B_n-_*_570_, with 4% precipitation at 130 °C and 5.7% precipitation at 165 °C (180 min). Based on the above results, the modification effect of KH570 on bagasse fiber was the best among the three silane coupling agents; thus, the lower the asphalt precipitation rate at high temperature, the better the high-temperature oil absorption performance of the fiber in the sample [46]. In Figure 4, *B_p_* is the unmodified bagasse fiber, *B_n_* is the bagasse fiber modified only by NaOH, *B_a_* is the basalt fiber, and *B_n-_*_550_, *B_n-_*_570_, and *B_n-_*_590_ are the bagasse fibers modified by NaOH combined with the silane coupling agents KH550, KH570, and KH590, respectively.

#### 4.1.2. Heat Stability

**Figure 5:**
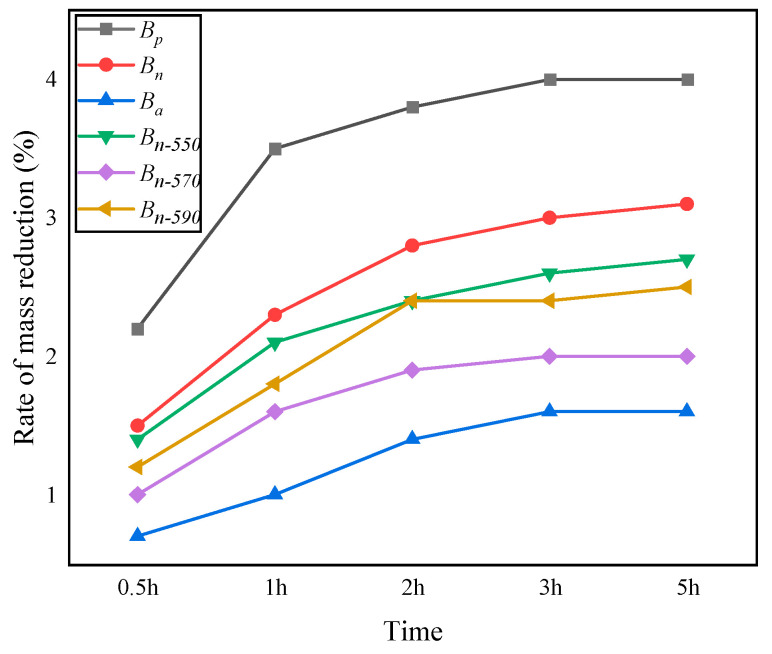
The change in mass loss over time.

The heat stability of bagasse fiber is related to its mass loss at high temperature: the less mass loss, the better the heat stability of the fiber. Figure 5 shows the mass loss of six kinds of fiber samples (placed in the oven at 200 °C for 5 h). All samples experienced the fastest mass loss in the first 2 h, and then the loss rate gradually slowed down. Basalt fiber (*B_a_*) has the smallest mass loss among the six samples, with a loss rate of only 1.6%, indicating that it can remain stable under long-term high-temperature conditions. The mass loss of unmodified bagasse fiber (*B_p_*) at 200 °C/0.5 h is as high as 2.9%, which is close to the mass loss of *B_n_* (3%) at 200 °C/3 h, indicating that the modification is conducive to improving the heat stability of the fiber. The mass loss values of *B_p_*, *B_n_*, *B_n-_*_550_, *B_n-_*_570_, and *B_n-_*_590_ after 200 °C/5 h were 5%, 3.2%, 2.7%, 2.0%, and 2.5%, respectively, indicating that NaOH combined with a silane coupling agent was more beneficial to the increase in the heat stability of bagasse fiber than that modified by NaOH alone. According to the heat loss performance of the three silane coupling agents, NaOH combined with *B_n-_*_570_ has the best effect on improving the heat stability of bagasse fiber.

#### 4.1.3. Hygroscopicity

**Figure 6:**
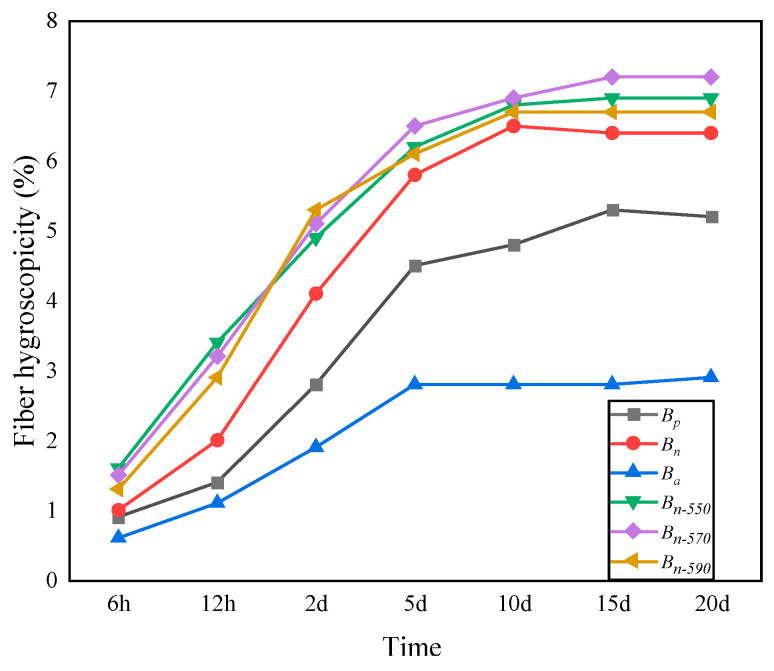
The change in hygroscopicity over time.

In hot and humid areas, fiber with high hygroscopic properties can easily absorb water and form into a group when stored, which is not conducive to the adhesion of fiber and asphalt during preservation and mixing [47]. Inorganic salt (such as Na_2_SO_4_, NaCl, etc.) in the water mist in a high-temperature and high-humidity salt area will corrode the fiber, as well as degrade the high-temperature stability and water erosion resistance of the asphalt mixture. As shown in Figure 6, with the increase in time, the water absorption rate of the fiber increased the fastest in the first five days, and then the speed gradually slowed. *B_n-_*_590_*, B_n-_*_550_, and *B_n-_*_570_ peaked on day 10 and then remained stable; *B_n_* peaked on day 13 and *B_p_* peaked on day 15. The difference among *B_n-_*_550_ (5.8%), *B_n-_*_570_ (5.5%), and *B_n-_*_590_ (5.2%) is within 10%, and the mean value of the three is 14.1% smaller than that of *B_n_* and 21.4% smaller than that of *B_p_*. The results show that the efficiency of removing hydrophilic (-OH) groups, hemicellulose, and pectin from the surface of bagasse fiber modified by a silane coupling agent combined with NaOH was higher than that modified by NaOH alone; a large amount of active hydrogen that can bind to water molecules was eliminated, and the hydrophilic ability of the fiber was inhibited. The results also show that there was no significant difference in the hygroscopic performance of bagasse fiber improved by different types of silane coupling agents. In addition, the moisture absorption rate of *B_n-_*_550_ was slightly higher than that of *B_n-_*_570_ and *B_n-_*_590_, which may be related to the certain polarity (affinity for water molecules) of the amino group contained in KH550.

#### 4.1.4. Surface Microscopic Analysis before and after Modification

**Figure 7:**
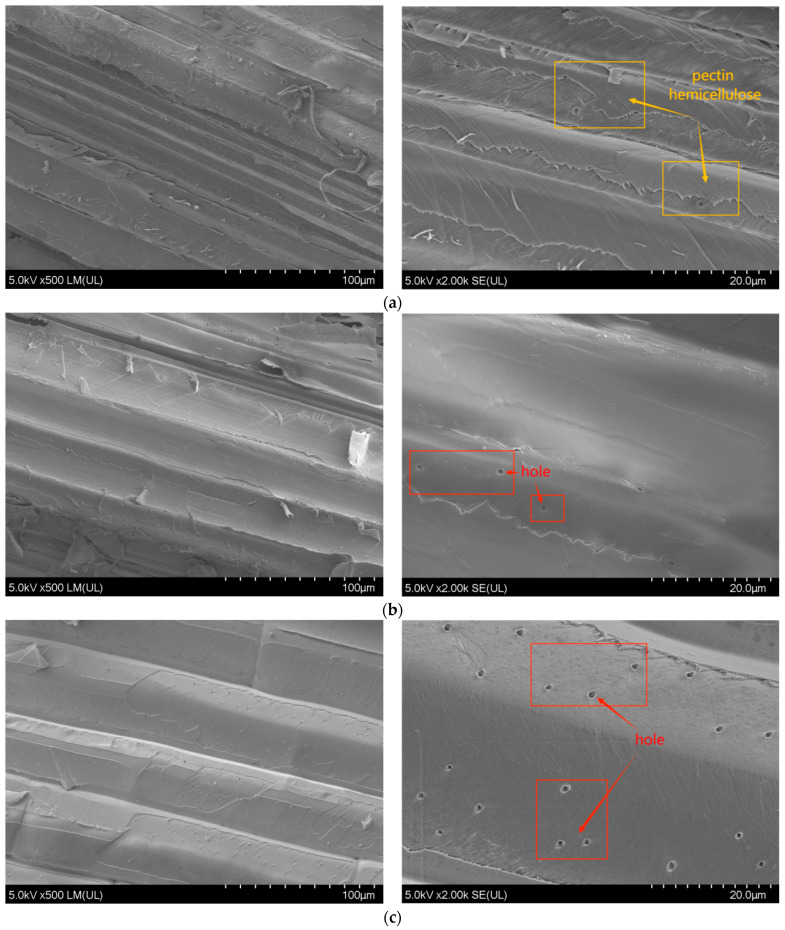

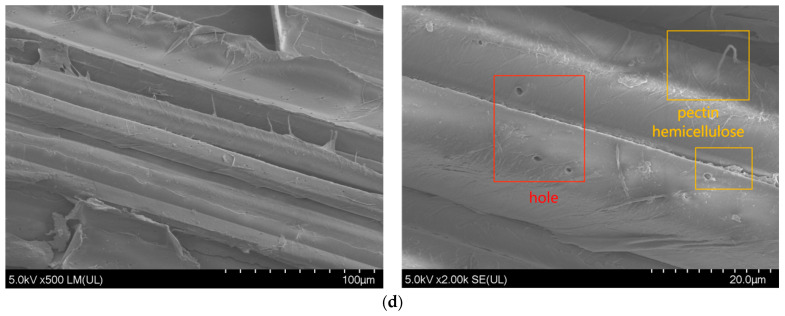
Surface micrographs of bagasse fiber treated with different modifiers. (**a**) Untreated; (**b**) treated with NaOH/KH550; (**c**) treated with NaOH/KH570; (**d**) treated with NaOH/KH590.

In order to further reveal the mechanism of improving the properties of bagasse fiber after modification, SEM was used to observe the surface of bagasse fiber before and after treatment with the NaOH-silane coupling agent. As shown in Figure 7a, the surface flatness of the original bagasse fiber was poor, there were many small molecular impurities (such as glue, a waxy layer, hemicellulose, etc.) covering the surface of the fiber, and the fibrous texture was fuzzy. After NaOH was combined with the silane coupling agent, the surface of the bagasse fiber gradually became smooth and bright, the texture between the fibers was clear, and the micropores on the surface of the fiber began to gradually appear after the impurities such as pectin and wax were removed. Compared with those shown in Figure 7b,d, the NaOH/KH570-treated bagasse fiber surface layer (Figure 7c) has the clearest texture, the largest number of micropores, and the largest specific surface area between fiber and asphalt (an increase in the specific surface area of the fiber can improve the adsorption of asphalt and improve the overall stability of the asphalt binder). The above results are consistent with those of the high-temperature oil absorption test, heat resistance test, and hygroscopicity test.

In summary, NaOH combined with KH570 is recommended as a modification scheme for bagasse fiber.

### 4.2. Analysis of Conventional Physical Properties and Storage Properties of Asphalt Binders in Different Erosion Environments

#### 4.2.1. General Physical Properties

**Figure 8:**
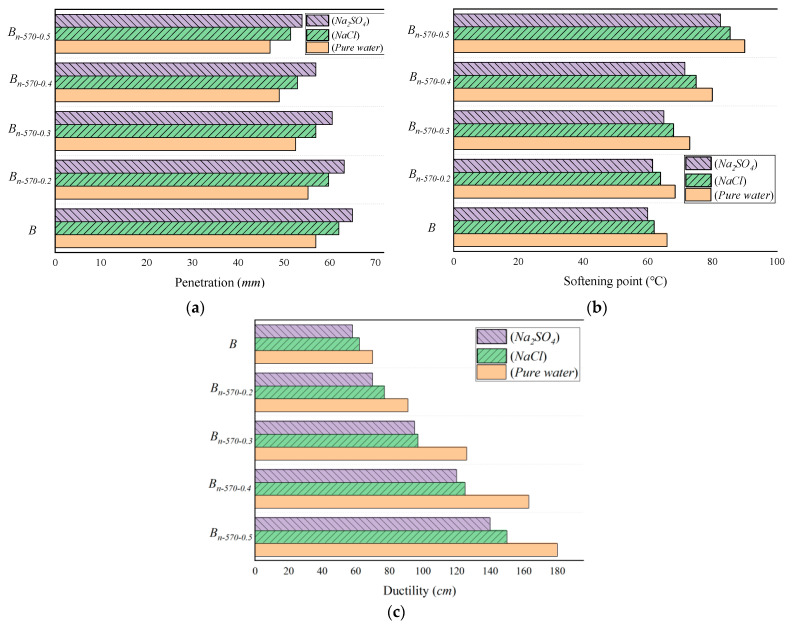
General physical properties of modified bagasse fiber asphalt binders under different erosion conditions. (**a**) Penetration; (**b**) softening point; (**c**) ductility.

As shown in Figure 8a, the changes in penetration with fiber content in different erosion environments are as follows: (1) With the same fiber content, the penetration of the asphalt binder after dry–wet cycling with clean water is higher than for NaCl and Na_2_SO_4_, indicating that a salt solution has a stronger ability to accelerate asphalt aging than pure water and has a greater impact on viscosity. (2) In the same erosion environment, with the increase in fiber content, the penetration of asphalt binder gradually decreases, and the viscosity and temperature stability increase. The decreasing trend of penetration is more obvious from *B_n-_*_570-0.2_ to *B_n-_*_570-0.4_, while the decreasing trend of *B_n-_*_570-0.4_ to *B_n-_*_570-0.5_ is slower than that of the former.

The change in the softening point of the asphalt binder with the increase in fiber content in different erosion environments is shown in Figure 8b. (1) When the fiber content is less than 0.2%, the softening point of the improved asphalt binder has little difference compared with that of the base asphalt binder. After the content exceeds 0.2%, the softening point increases gradually (the peak growth rate is from 0.4% to 0.5%). That is, the improvement in the softening point of the asphalt binder needs to be sensitive after the fiber content reaches a certain level, and it is not helpful to improve the high-temperature stability of the asphalt binder if the fiber content is less than 0.2%. (2) The softening point of the modified bagasse asphalt binder with the same dosage varies in different erosion environments. When the erosion environment contains a Na_2_SO_4_ solution, the softening point is smaller than that of an NaCl solution and pure water, but the difference between the three decreases gradually with the increase in fiber content, indicating that the increase in fiber content can reduce the sensitivity of the asphalt softening point to different erosion environments.

The change in ductility with fiber content in different erosion environments is shown in Figure 8c. (1) With the increase in fiber content, the ductility of the asphalt binder in different erosion environments tends to increase, and its resistance to low-temperature cracking is also improved. (2) The ductility of the asphalt binder eroded by Na_2_SO_4_ is smaller than that of NaCl and pure water.

In summary, Na_2_SO_4_ solution causes the greatest erosion damage to the asphalt binder; this is the worst erosion environment. Considering the improvement effect of different fiber contents on various indexes, the best effect is achieved when the content is 0.5%. In the worst erosion environment (Na_2_SO_4_ solution), the penetration, softening point, and ductility of 0.5% asphalt mortar with fiber content increased by −16.9%, 37.5%, and 105.2%, respectively, compared with the base asphalt (B). (Note: B stands for base asphalt; *B_n-_*_570-0.2_, *B_n-_*_570-0.3_, *B_n-_*_570-0.4_, and *B_n-_*_570-0.5_ represent the asphalt binder with 0.2%, 0.3%, 0.4%, and 0.5% bagasse fiber contents, respectively, modified by NaOH/KH570.)

#### 4.2.2. Storage Performance

According to Fang et al. (2013) [48], the filament or partly network-like structure formed in the modified asphalt system is beneficial to improving the hot storage stability, and it is necessary to study the effect of the modified asphalt fiber content on its storage stability. As shown in Table 8, the segregation degree of each asphalt binder sample was divided into four stages with the increase in storage time. Stage 1 is 0–3 h; each asphalt sample has no obvious segregation phenomenon, the whole sample is in a homogeneous state, and the difference in the softening point between different samples is not substantial. Stage 2 is 3–6 h; the separation speed of the modifier and fiber is accelerated, and the softening point difference increases rapidly. Stage 3 is 6–12 h; the segregation speed falls back and the difference decreases. Stage 4 is 12–24 h; the aggregate of the modifier and fiber at the bottom of the segregation tube has mostly been completed, the softening point difference has undergone very little change (<0.2 °C), and the asphalt tends to be stable inside. In stage 4, the difference in the softening point of B is 1.26 times that of *B_n-_*_570-0.2_, 1.75 times that of *B_n-_*_570-0.3_, 2.32 times that of *B_n-_*_570-0.4_, and 2.63 times that of *B_n-_*_570-0.5_, indicating that *B_n-_*_570-0.5_ has the smallest softening point difference and the lowest degree of segregation. According to the test results for the softening point difference, 0.5% modified bagasse fiber has the strongest ability to adsorb and aggregate asphalt at high temperatures, which can improve the overall stability of asphalt binder.

### 4.3. Rheological Property Analysis of Bitumen with Different Bagasse Fiber Contents in a High-Temperature and High-Humidity Salt Environment

#### 4.3.1. Rotational Viscosity

**Figure 9:**
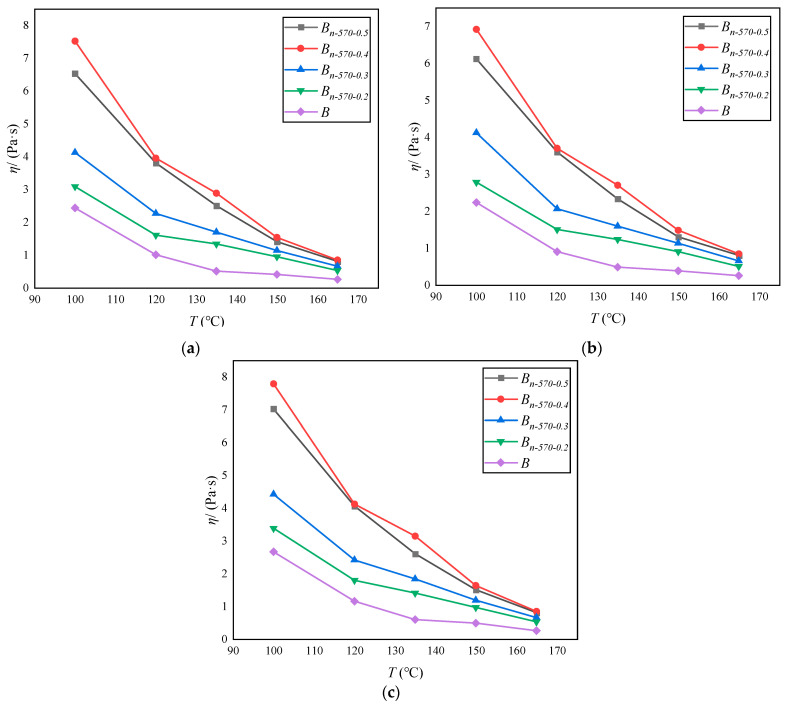
Rotational viscosity of asphalt binder under different erosion conditions: (**a**) 10% NaCl; (**b**) 10% Na_2_SO_4_; (**c**) pure water.

As shown in Figure 9, at the same temperature, the viscosity of asphalt in different erosion environments increased with the increase in the content of modified bagasse fiber, and the viscosity reached its peak when the content was 0.5%. The greatest increase occurred when the content increased from 0.3% to 0.4%, but the increase rate decreased rapidly when the content increased from 0.4% to 0.5%, indicating that the high fiber content also causes adverse effects on the viscosity of asphalt, which is similar to the test results of Ahmed et al. (2022) [10] and Kou et al. (2019) [49]. When the fiber content is close to the optimal content, the asphalt sample stability may remain unchanged or may gradually decline.

In addition, the viscosity of the modified bagasse fiber asphalt with different dosages shows a nonlinear attenuation trend with the increase in temperature. When the temperature is less than 150 °C, the influence of different fiber contents on the viscosity is more obvious, and the difference decreases sharply when the temperature is higher than 150 °C, indicating that the stability of asphalt is very sensitive to high-temperature environments. Taking pure water as an example, the viscosity of *B_n-_*_570-0.3_ at 165 °C is only 35% of that at 135 °C, and 15% of that at 100 °C.

**Figure 10:**
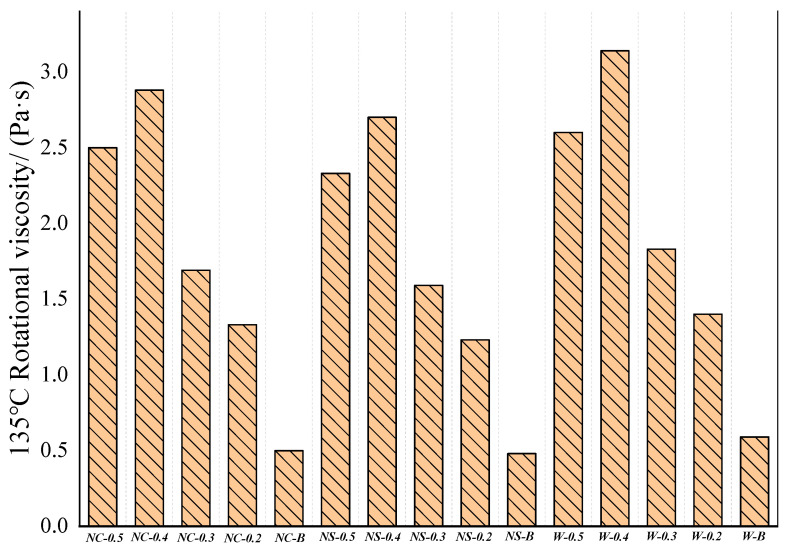
Rotational viscosity at 135 °C.

Figure 10 shows the viscosity at 135 °C. After dry–wet cycle treatment with water, NaCl, and Na_2_SO_4_, the viscosity of *B_n-_*_570-0.5_ was 3.14 Pa·s, 2.88 Pa·s, and 2.7 Pa·s. That is, the viscosity after water erosion was 9.0% and 16.3% higher than NaCl and Na_2_SO_4_, respectively, indicating that the salt solution has a greater impact on the overall stability of asphalt than clean water. The Na_2_SO_4_ solution is more erosive than the NaCl solution. In the process of dry–wet cycling, the salt solution enters the space of different sizes inside the glue. As the salt is dissolved at normal temperature (20 °C) and the salt is precipitated after the water evaporates at high temperature (65 °C), the salt crystals remaining in the space puncture the asphalt film on the surface of the mineral powder particles, affecting the high-temperature rheological properties of the asphalt. (Note: In Figure 10, NC means NaCl, NS means Na_2_SO_4_, W means pure water, 0.5 means *B_n_*_-570-0.5_, and 0.5-W means *B_n_*_-570-0.5_ treated with pure water dry–wet cycles.)

#### 4.3.2. Dynamic Shear Rheological Test

**Figure 11:**
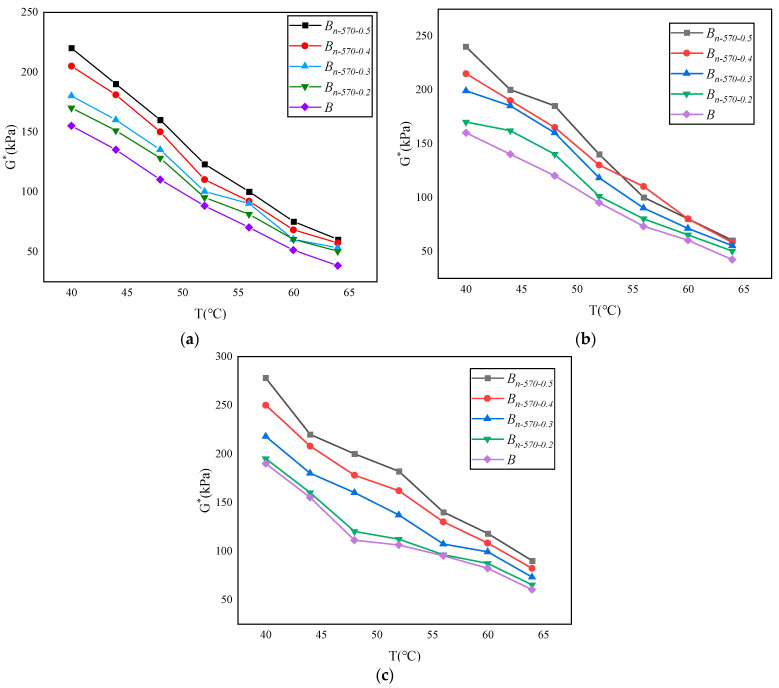
Variation in complex shear modulus (G*) with temperature in different erosion environments: (**a**) 10% Na_2_SO_4_; (**b**) 10% NaCl; (**c**) pure water.

**Figure 12:**
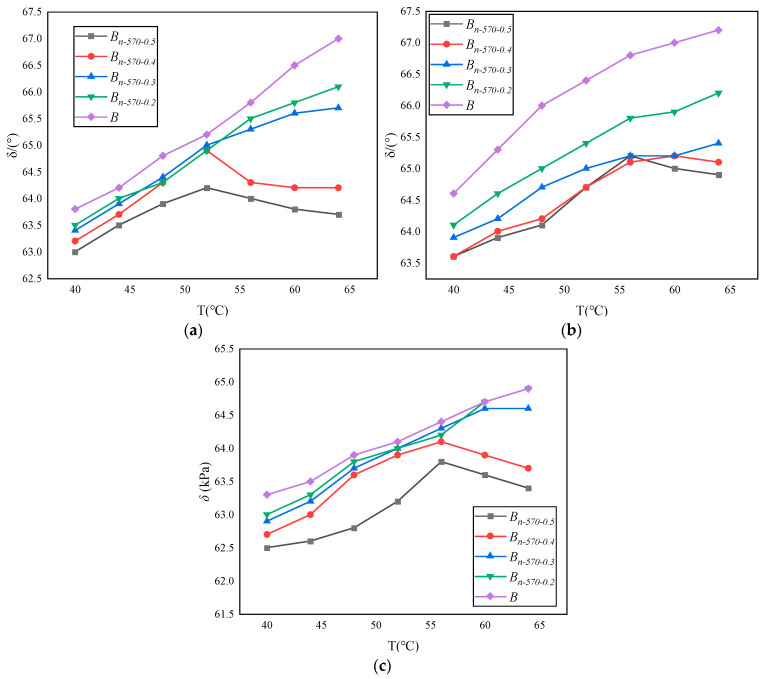
Variation in phase angle (δ) with temperature in different erosion environments: (**a**) 10% Na_2_SO_4_; (**b**) 10% NaCl; (**c**) pure water.

**Figure 13:**
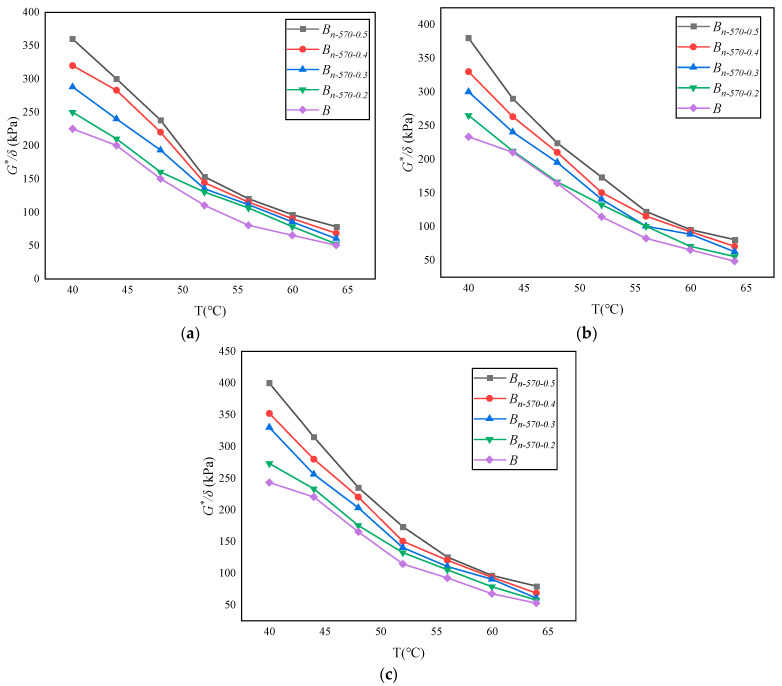
Variation in rutting factor (G*/δ) with temperature in different erosion environments: (**a**) 10% Na_2_SO_4_; (**b**) 10% NaCl; (**c**) pure water.

The complex shear modulus G* and phase angle *δ* in the dynamic shear rheological tests are used to characterize the viscosity (non-recoverable deformation) and elasticity (recoverable deformation) of asphalt materials, respectively, together characterizing the high-temperature and fatigue resistance of asphalt materials. G* is a measure of the total stress when the material is subjected to repeated shear deformation. The larger the value of G*, the stronger the deformation resistance of the asphalt binder. *δ* is a relative index used to measure the ability of recoverable deformation and non-recoverable deformation. The larger the value of *δ*, the larger the viscous composition of the binder. The rutting factor (G*/sin *δ*) is a technical index used to evaluate the high-temperature characteristics of the asphalt binder, reflecting the ability of the binder to resist permanent deformation. Therefore, in SHRP specifications, the larger the value of G*/sin *δ*, the smaller the flow deformation of the asphalt binder, and the more conducive it is to resisting rutting. The changes in G*, *δ*, and G*/sin *δ* of the modified bagasse fiber asphalt binder with temperature under different types of dry–wet cycle conditions are shown in Figure 11, Figure 12 and Figure 13.

1. Under the same erosion environment and test temperature, the complex shear modulus (G*) and rutting factor (G*/sin *δ*) of *B_n-_*_570-0.5_ are the largest, and the phase angle *δ* is the smallest. The results show that the increase in modified bagasse fiber content is positively correlated with the improvement in asphalt deformation resistance under the same external conditions.
2. Under the same fiber content and test temperature, Na_2_SO_4_ has the greatest inhibition on the complex shear modulus (G*) of asphalt. Taking *B_n-_*_570-0.5_ in Figure 11 as an example, the values of G* in pure water, NaCl, and Na_2_SO_4_ are 278.3 kPa, 182.4 kPa, and 138 kPa at 40 °C; 241.5 kPa, 140.9 kPa, and 104.2 kPa at 52 °C; and 221.8 kPa, 123.5 kPa, and 102.3 kPa at 56 °C. In addition, when the temperatures are 40 °C, 52 °C, and 56 °C, the absolute values of the difference between the complex shear modulus of *B_n-_*_570-0.5_ eroded by NaCl and Na_2_SO_4_ are 44.4, 36.7, and 21.2, respectively, indicating that the difference between the complex shear modulus (G*) in NaCl and Na_2_SO_4_ gradually shrinks with the increase in temperature. That is, the increase in temperature weakens the difference in the erosion effect of different types of salt erosion on the asphalt binder.
3. When the erosion environment is the same but the temperature is different, there are two trends in phase angle change. One is that the phase angle increases with the increase in temperature, as shown in Figure 12. *B*, *B_n-_*_570-0.2_, and *B_n-_*_570-0.3_ indicate that the effect of a low fiber content on inhibiting the “asphalt flow” is not obvious, and the asphalt mortar gradually changes from a solid to a fluid, similar to a Newtonian fluid, with the increase in temperature. The other trend is that there is a stage of “basically unchanged or slowly decreasing” after the phase angle reaches the peak with the increase in temperature, such as *B_n-_*_570-0.4_ and *B_n-_*_570-0.5_ in Figure 12, which indicate that the distribution of fibers in the asphalt is sufficient. The bridge effect is used to increase the friction between asphalt molecules and, to some extent, to restrain the flow deformation of asphalt under high-temperature shear force. The above trends in phase angle change are similar to the study of Cui et al. (2022) [45] (that is, the phase angle of base asphalt increases with increasing temperature, while that of fiber-asphalt increases first and then slightly decreases), but the peak temperature of the asphalt phase angle is 10 °C higher than modified bagasse fiber asphalt (56 °C), which may be related to asphalt materials with stronger adhesion being selected.
4. However, if the temperature continues to rise, the phase angle will return to an increasing state after a short decrease, the fiber function will be sharply weakened, and the overall deformation resistance of the asphalt will also decline rapidly (Figure 13). The complex shear modulus (G*) and rutting factor (G*/sin *δ*) will eventually approach 0.

#### 4.3.3. Analysis of MSCR Test Results

As can be seen from Figure 13, under the same test conditions (erosion type and fiber content), the creep recovery rates of the asphalt binder are *R*_3.2_ < *R*_0.1_ and *J_nr_*_3.2_ > *J_nr_*_0.1_, indicating that the greater the stress, the more unrecoverable deformation of the asphalt binder. Under the same fiber content, the order of the *R* value is water > NaCl > Na_2_SO_4_, and the order of *J_nr_* is Na_2_SO_4_ > NaCl > water. That is, Na_2_SO_4_ has the strongest erosion ability in respect of asphalt mortar, followed by NaCl and, finally, pure water.

**Figure 14:**
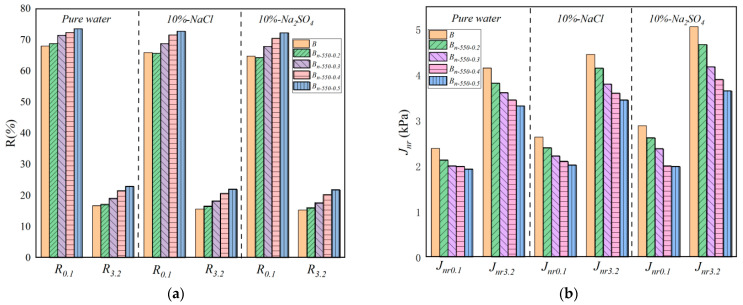
The results of the MSCR experiment. (**a**) Creep recovery rate; (**b**) unrecoverable deformation.

As shown in Figure 14a, compared with base asphalt (*B*), the *R*_0.1_ value of asphalt binder mixed with 0.2%, 0.3%, 0.4%, and 0.5% modified bagasse fiber in pure water increased by 1.26%, 5.15%, 9.47%, and 11.57%; in NaCl, the value increased by −0.3%, 4.4%, 8.65%, and 10.47%; and, in Na_2_SO_4_, the value increased by −0.7%, 4.78%, 8.23%, and 9.8%. Under the same circumstances, compared with base asphalt (*B*), *R*_3.2_ in pure water increased by 5.06%, 15.79%, 32.9%, 43.42%; in NaCl, it increased by 4.5%, 17.42%, 30.9%, and 41.94%; and, in Na_2_SO_4_, it increased by 2.3%, 14.46%, 29.52%, and 37.95%, respectively.

As shown in Figure 14b, compared with base asphalt (*B*), the *J_nr_*_0.1_ value of asphalt binder mixed with 0.2%, 0.3%, 0.4%, and 0.5% modified bagasse fiber in pure water decreased by 9.0%, 17.36%, 20.5%, and 32%; in NaCl, it decreased by 8.74%, 15.59%, 20.15%, and 26.19%; and, in Na_2_SO_4_, it decreased by 10.5%, 15.97%, 19.39%, and 20.91%. Under the same circumstances, compared with base asphalt (*B*), *J_nr_*_3.2_ decreased by 7.95%, 13%, 16.87%, and 20% in pure water; decreased by 6.79%, 14.60%, 19.12%, and 22.46% in NaCl; and decreased by 7.71%, 16.99%, 22.92%, and 27.86% in Na_2_SO_4_.

In summary, (1) the changes in *R* and *J_nr_* are large, from 0.2% to 0.4% of the fiber content increase, and narrow, from 0.4% to 0.5%. The fiber performance is the best when the fiber content is 0.5% (maximum *R*-value and minimum *J_nr_*-value). (2) The modified bagasse fiber asphalt samples with different mass fractions have certain differences at 0.1kPa and 3.2kPa. When the fiber content is less than 0.2, the difference between the *R*_0.1_ of *B_n-_*_570-0.2_, *R*_0.1_ of *B_n-_*_570-0.3_, and *R*_3.2_ of *B_n-_*_570-0.2_ in pure water, NaCl, and Na_2_SO_4_ and the base asphalt is less than 5% (in statistical analysis, a change within 5% is considered to indicate no significant difference). When the fiber content is greater than 0.3%, the modified bagasse asphalt gradually exhibits better creep ability than the base asphalt, indicating that a small amount of bagasse fiber does not significantly improve the creep deformation ability of asphalt. (3) Under the same stress level, the creep recovery rate (*R*) of the asphalt samples can be increased with the increase in fiber content, and the non-recoverable creep compliance (*J_nr_*) can be reduced. In other words, the addition of modified bagasse fiber can inhibit the development of unrecoverable deformation of asphalt binder and improve the elastic recovery ability of asphalt.

#### 4.3.4. Analysis of Linear Amplitude Scanning Test (LAS) Results

**Figure 15:**
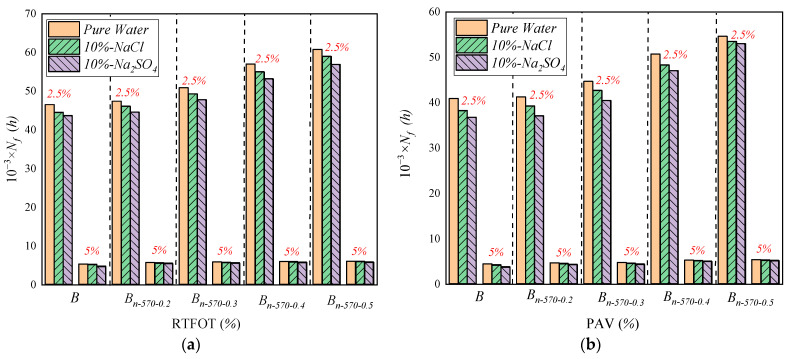
Fatigue life of asphalt samples after short-term and long-term aging. (**a**) Short-term aging; (**b**) long-term aging.

As shown in Figure 15, when the strain is 5%, the fatigue life of each asphalt sample decreases by 80–90% compared with that when the strain is 2.5%. The fatigue life of asphalt binder after long-term aging is reduced by 10~25% compared to that of asphalt binder after short-term aging, indicating that the fatigue life of asphalt can be reduced by increasing the strain and aging degree, and the change in strain has a more significant effect on fatigue life [8].

Modified bagasse fiber can improve the short-term and long-term fatigue resistance of asphalt in LAS testing, but the performance of the 2.5% strain is different from that of the 5% strain. Figure 15a shows the performance of the asphalt binder after short-term aging. Compared with base asphalt (*B*), under 2.5% strain, the fatigue life of *B_n-_*_570-0.5_ treated with NaCl and Na_2_SO_4_ increases by 14,510 and 13,190, and the fatigue life of *B_n-_*_570-0.5_ treated under 5% strain increases by 870 and 1090. Figure 15b shows the performance of asphalt binder after long-term aging. Compared with base asphalt (*B*), under 2.5% strain, the life of *B_n-_*_570-0.5_ treated with NaCl and Na_2_SO_4_ increases by 15,240 and 16,230, and the life of *B_n-_*_570-0.5_ treated with 5% strain increases by 1070 and 1440, respectively. The results show that the improvement in the fatigue life of *B_n-_*_570-0.5_ under 5% strain is only 10% of that under 2.5% strain. That is, the greater the strain, the weaker the effect of fiber incorporation on improving the fatigue life of asphalt binder.

For the same amount of modified bagasse fiber, the erosion effect of the salt solution on the fatigue life of asphalt is greater than that of pure water. Taking *B_n-_*_570-0.3_ in Figure 15a as an example, after short-term aging, compared with pure water, the fatigue lives under 2.5% strain and 5% strain are reduced by 1600 and 80 in NaCl, and by 2500 and 120 in Na_2_SO_4_. Similarly, after long-term aging, the fatigue lives under 2.5% strain and 5% strain are reduced by 1900 and 110 in NaCl, and by 2230 and 210 in Na_2_SO_4_. That is, salt solution erosion is more harmful to asphalt than pure water, and Na_2_SO_4_ in salt solution is more corrosive than NaCl, which has a greater impact on asphalt fatigue life. It is worth noting that, under the condition of short-term aging, the life gaps of *B*, *B_n-_*_570-0.2_, *B_n-_*_570-0.3_, *B_n-_*_570-0.4_, and *B_n-_*_570-0.5_ after NaCl and Na_2_SO_4_ erosion under 2.5% strain are 2500, 2000, 1400, 700, and 500, and under 5% strain are 470, 430, 320, 130, and 120, respectively. Similarly, after long-term aging, the life differences under 2.5% strain are 2370, 2170, 1480, 670, and 490, and under 5% strain are 450, 210, 190, 100, and 70. The above difference indicates that the sensitivity of the asphalt binder to the two different salt solutions (NaCl and Na_2_SO_4_) decreases with the increase in the content of modified bagasse fiber, and the difference is basically negligible when the content is >0.4%.

#### 4.3.5. Four-Point Bending Fatigue Life Analysis of Modified Bagasse Fiber Asphalt Mixture

The fatigue life test results and fatigue life models of the modified bagasse fiber asphalt mixture under different strain levels are given in Table 9. It can be seen that the fatigue life of the asphalt mixture decreases rapidly with the increase in strain, which is similar to the change in the asphalt binder [45]. Taking NaCl as an example, the lives of *B_n-_*_570-0.3_ and *B_n-_*_570-0.4_ in *N_f-450_* are 2.37 times and 11.65 times that of *N_f-750_*, and 4.5 times and 13.2 times that of *N_f-1050_*, respectively, because the increase in strain accelerates the growth of capillary cracks in the asphalt mixture. The stability between the asphalt binder and the aggregate is destroyed, resulting in the stiffness of the mixture rapidly decreasing to the failure condition (less than 50% of the original stiffness). In pure water, when the fiber content is greater than 0.3%, under high-strain conditions, such as *N_f-1050_*, the fatigue life is only increased by about 10% on average for each 0.1% increase in fiber, and a similar situation also occurs in NaCl (increased by about 12% on average) and Na_2_SO_4_ (increased by about 13% on average). Under low-strain conditions, such as *N_f-450_*, when the fiber content exceeds 0.3%, the fatigue life increases by 22% in pure water, 20% in NaCl, and 22% in Na_2_SO_4_, on average, for each 0.1% increase in fiber content. This indicates that the high-strain condition reduces the effect of high-fiber-content asphalt on improving the fatigue life of the mixture.

In addition, under the same fiber content and stress conditions, the order of the impact of the erosion environment on fatigue life is as follows: clean water < NaCl < Na_2_SO_4_, which means the erosion environment of Na_2_SO_4_ causes the greatest damage to the asphalt mixture. The fatigue life growth rates are different at different fiber dosage stages. Taking *N_f-_*_450_ as an example, the fatigue life of the mixture under the erosion of pure water, NaCl, and Na_2_SO_4_ increases by 3359, 2968, and 2704 for the fiber dosage from 0 to 0.2%, increases by 22,912, 20,081, and 18,904 for 0.2% to 0.3%, and increases by 10,357, 9358, and 8723 for 0.3% to 0.4%, respectively. That is, a fiber content of ≤0.2% cannot effectively inhibit the development of capillary cracks in the asphalt mixture, and it provides little help to the fatigue life growth of the asphalt mixture. A more obvious improvement effect can be obtained when the fiber content is greater than 0.3%, and the greater the fiber content, the smaller the difference in the fatigue life of the asphalt mixture in the two salt solutions. Based on the above fatigue life results, the asphalt mixture with 0.5% fiber content has the longest life under different erosion and strain conditions. Compared with Shafabakhsh et al. (2014) [44], the fatigue life (*N_f-_*_750_ and *N_f-_*_1050_) of *B_n_*_-570-0.5_ approaches asphalt modified with 4%Nano + 5%SBS, which means that SBS materials may be replaced by bagasse fiber in the future, based on the economy and availability of bagasse fiber.

### 4.4. Analysis of the Erosion Mechanism of Modified Bagasse Fiber Asphalt Binder and Mixture in a High-Temperature and High-Humidity Salt Environment

#### 4.4.1. Analysis of the Aging Mechanism of Pure Water on Asphalt Binder and Asphalt Mixture

The southern coastal areas of Guangdong have high average temperatures and heavy rainfall. High temperatures will soften the asphalt binder and produce a large number of microvoids. High humidity will make the moisture remaining between the asphalt mixture surface layer and the base layer slowly dip into the internal void of the asphalt binder, and cause some strong hydrophilic groups (-OH, -CHO, -COOH, -NH2, etc.) at the interfaces between asphalt and mineral, asphalt and modifier, etc., to be dissolved, absorbed, and lost via water, weakening the interface bonding force between the asphalt and the aggregate. This means that the stability of the combination of asphalt film and aggregate is reduced, the asphalt film peels off from the aggregate, and, ultimately, the overall strength of the pavement structure is reduced. In addition, under the repeated action of the dynamic load of the wheel, the water trapped in the pores of the asphalt mixture will continuously generate dynamic water pressure and vacuum re-compression suction, so that the water gradually permeates into the interface between the asphalt film and the aggregate, reducing their viscosity and gradually peeling the asphalt film from the aggregate, as well as causing multiple micro-cracks (such as the angular asphalt film of the aggregate covering a thin position) and constant expansion. Finally, diseases such as looseness, pushing, and rutting are caused.

#### 4.4.2. Analysis of the Erosion Mechanism of Salt Solution on Asphalt Binder and Mixture

Under the erosion of the dry–wet cycle, inorganic salt produces crystallization with the evaporation of water, and crystals are coated on the surface of the aggregate, reducing the adhesion between the asphalt and the aggregate. In addition, after water evaporation, the volume of inorganic salt crystals expands, and the increased volume leads to expansion and cracking inside the asphalt mixture due to its limited void space. Under the combined action of the cyclic expansion pressure and the permeability pressure in the pores caused by the evaporation of high-temperature water, new micro-cracks will constantly develop inside the asphalt mixture. The increasing number of micro-cracks accelerates the intrusion of inorganic salts into the asphalt and aggregate interface, producing larger cracks, resulting in a vicious cycle.

Table 10 shows the surface tension of each erosion solution, and the surface free energy of *B_n-_*_570-0.4_. Through calculation, the comprehensive stripping work of the asphalt–aggregate interface in various erosion environments is obtained.

**Figure 16:**
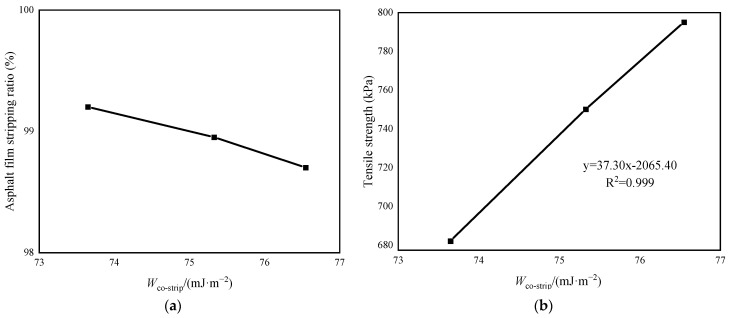
Relationship curve between asphalt film stripping ratio, tensile strength, and comprehensive strip-ping work. (**a**) Asphalt film stripping ratio; (**b**) tensile strength.

The relationships between the comprehensive stripping work of the sample and the asphalt film stripping ratio, and the comprehensive stripping work and the tensile strength of the asphalt film, both show a trend of synchronous monotonic change (shown in Figure 16). That is, the larger the comprehensive stripping work of the erosion environment, the smaller the stripping ratio of the asphalt film on the failure surface, and the higher the residual tensile strength of the asphalt–aggregate sample. Combined with the conventional physical and rheological properties of the asphalt binder, the fatigue life of the asphalt mixture, and comprehensive stripping work, the order of the erosion capacity is Na_2_SO_4_ > NaCl > pure water.

#### 4.4.3. Analysis of the Durability Mechanism of Modified Bagasse Fiber/Nano-TiO_2_ to Enhance Asphalt Binder and Mixture in a High-Temperature and High-Humidity Salt Environment

Bagasse fiber has a large specific surface area, which plays the role of absorbing asphalt, bridging aggregate, and strengthening the structure in the asphalt binder and the asphalt mixture. The addition of fiber reduces the migration degree of free asphalt in the asphalt mixture, increases the proportion of structural asphalt, and improves the overall strength and stability of the asphalt binder and the asphalt mixture. An alkali solution/silane coupling agent can more effectively decompose impurities such as hemicellulose and pectin in bagasse, increase the purity of cellulose, and weaken the hydrophilic ability of fiber, as well as improve the adsorption capacity of fiber by increasing the surface structure depth and gap. Nano-TiO_2_ can fill the microscopic defects of the asphalt binder, reduce its deterioration degree in a high-humidity salt environment, and improve the residual resistance to salt erosion after aging of the binder. Therefore, modified bagasse fiber combined with nano-TiO_2_ is feasible for enhancing the durability of the asphalt binder and mixture in a high-temperature and high-humidity salt environment.

**Figure 17:**
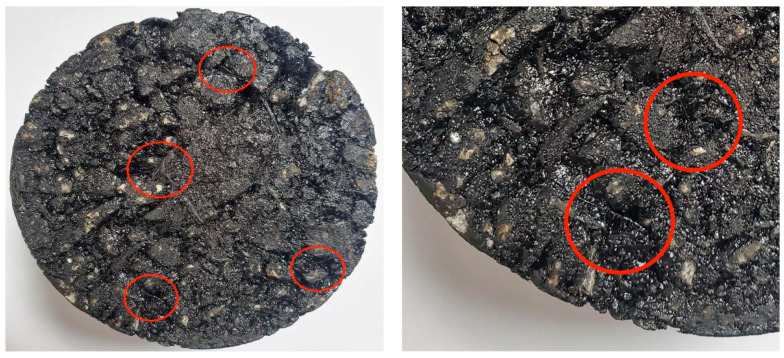
Surface of asphalt mixture with evenly distributed fibers.

**Figure 18:**
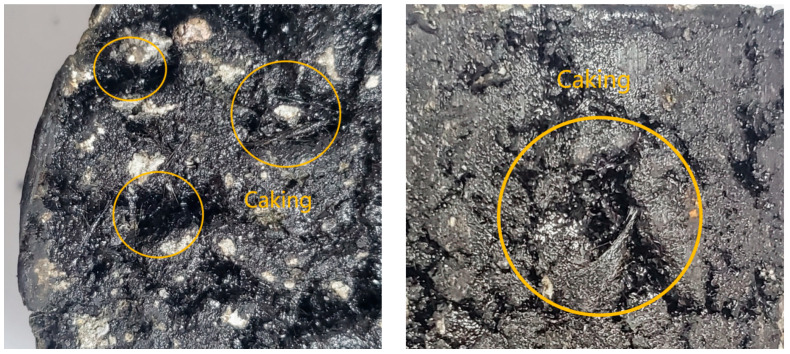
Asphalt mixture surface with uneven fiber distribution and high fiber agglomeration rate.

Modified bagasse fiber has a stable state, which helps to reduce the amount of water and inorganic salt solution entering the asphalt film and aggregate interface through the existing gaps. However, according to the above test results, if the fiber content is too low (≤0.2%), it is relatively dispersed in the asphalt binder and mixture, and it cannot fully transfer the internal stress and play the role of reinforcement anchorage when subjected to external loads. When the content is 0.3–0.4%, the dispersion of the fiber is gradually improved, forming a uniformly bonded, dense mesh and playing the role of reinforced anchorage, helping the mixture to better diffuse the internal stress and inhibit the generation and expansion of micro-cracks (as shown in Figure 17). When the fiber content increased from 0.4% to 0.5%, some performance improvement rates began to slow down, indicating that, when the fiber content approached or exceeded the optimal content, the fiber could not be effectively distributed, the clumping rate increased, the clumped fiber formed a weak layer inside the asphalt mixture specimen, and the strengthening trend of “reinforcement” and “bridge” became slower (as shown in Figure 18). Although the growth rate of each index slowed down from 0.4% to 0.5%, the overall stability of 0.5% asphalt samples was still better than that at 0.4%.

Based on the above tests and analyses, it is recommended to adopt the scheme of 0.5% modified bagasse fiber to improve the asphalt binder in a high-temperature and high-humidity salt environment.

## 5. Conclusions

Based on the lack of durability of traditional asphalt pavement in a high-temperature and high-humidity salt environment, a modified bagasse fiber/nano-TiO_2_ asphalt improvement scheme was proposed. Firstly, the optimum modification method of bagasse fiber was determined via a series of performance tests and microscopic analysis. Secondly, the conventional physical properties and rheological properties of modified bagasse fiber asphalt in different erosion environments and different fiber contents were investigated, and the best improvement scheme for bagasse fiber in the worst erosion environment was determined. The results are as follows:

1. Compared with the sole use of NaOH, a NaOH/silane coupling agent can more effectively remove the surface impurities of the original bagasse fiber, increase the specific surface area of the fiber and asphalt, and improve the stability of fiber and asphalt bonding. However, there is little difference in the improvement in the moisture absorption performance of bagasse fiber with different types of silane coupling agent.
2. Compared with the silane coupling agents KH550 and KH590, bagasse fiber modified with NaOH combined with KH570 showed better high-temperature oil absorption and heat stability, as well as greater compatibility with base asphalt.
3. The Na_2_SO_4_ solution provides the worst erosion environment, and the order of erosion capacity is Na_2_SO_4_ > NaCl > pure water. The increase in test temperature and modified fiber content can weaken the sensitivity of asphalt binder to different erosion environments.
4. When the fiber content is less than 0.2%, a uniform and overlapping mesh structure cannot be formed in the asphalt binder, and the improvement effect is not obvious. When the fiber content is greater than 0.5%, the clumping rate will increase, and the clumped fiber will form a weak layer inside the asphalt mixture specimen, weakening the “reinforcement” and “bridging” roles.
5. The 0.5% modified bagasse fiber/nano-TiO_2_ asphalt binder and the mixture have the best effect in a high-temperature and high-humidity salt environment. In the worst erosion environment, the penetration, softening point, creep recovery rate *R*_3.2_, non-recoverable creep compliance *J_nr_*_3.2_, and PAV(5% strain)-fatigue life of *B_n-_*_570-0.5_ were increased by −16.9%, 37.5%, 37.95%, −27.86%, and 38.30%, respectively, compared with the base asphalt binder (*B*). In addition, the four-point flexural fatigue life of the *B_n-_*_570-0.5_ was 169.2% higher than that of the ordinary mixture without fiber.

## Figures and Tables

**Table 1:** Properties of base asphalt.

Tested Property		Unit	Test Value	Standard Value	Standard
Penetration index		-	0.62	−1.5 ± 1.0	T0604
Softening point		°C	48	≥44	T0606
Flash point		°C	285	≥260	T0611
Dynamic viscosity	135 °C	Pa·s	2.5	≤3	T0739
Penetration	15 °C, 100 g, 5 s	0.1 mm	44	-	T0604
	25 °C, 100 g, 5 s		85	80–100	
	30 °C, 100 g, 5 s		162	-	
Ductility	10 °C, 5 cm/min	cm	29	≥20	T0605
	15 °C, 5 cm/min	cm	>170	≥100	
After RTFOT	Quality loss	%	0.09	≤1	T0610
	Residual ductility (5 °C)	cm	70.1	≥65	
	Residual ductility (15 °C)	cm	16.9	≥15	
	Penetration ratio (25 °C)	%	66.4	≥57	

**Table 2:** Properties of mineral filler.

Tested Property		Unit	Test Value	Standard Value	Standard
Apparent density		g/cm^3^	2.75	≥2.5	T0352
Water content		%	0.2	≤1	T0332
Hydrophilic coefficient		-	3	<1	T0353
Particle size	<0.6 mm	%	99.7	100	T0351
	<0.3 mm	%	99.5	-	
	<0.15 mm	%	99.2	90–100	
	<0.075 mm	%	88.1	75–100	

**Table 3:** Fiber performance index.

Tested Property	Diameter/μm	Length/mm	Water Content/%	Oil Absorption Rate	Heat Resistance (210 °C, 2 h)/%	Melting Point/°C	PH	Ash Content	Tensile Strength/MPa
Bagasse fiber	300	4–6	4.5	7.4	3.8	176	6.9	18	172
Standard	-	≤6	<5	≥5	≤6 (no combustion)	4.2	6.5–8.5	18 ± 5	-

**Table 4:** Technical indicators of chlorine salt.

Ingredients	NaCl	Water	K	Mg, Ca	Water Insoluble	Density (g/cm^3^)	Solubility/g	Dry Weight Loss
Percentage	≥99.5%	<0.5%	≤0.02	0.01	≤0.05	2.189	35	0.5%

**Table 5:** Technical indicators of sodium sulfate.

Ingredients	Na_2_SO_4_	Cl	K, Ca, Fe	Water Insoluble	Density (g/cm^3^)	Solubility/g (20 °C)	Scorch Weightlessness
Percentage	≥99.5%	≤0.1%	≤0.2%	≤0.05	2.702	20	0.2%

**Table 6:** Physical parameters of nano-TiO_2_.

Name	Appearance	Average Particle Size	Purity	Density (g/cm^3^)	Specific Surface Area (m^2^/g)	PH	Drying Weight Loss (%)	Scorch Weight Loss (%)	Crystalline
TiO_2_	White powder	20 nm	99.99	3.8/4.2	68–90	6–8	0.5	1.0	De-titania/rutile

**Table 7:** Performance indicators of silane modifiers.

Name	Chemical Formula	Boiling Point (760 mmHg)/°C	Density (ρ20), g/cm^3^	Flash Point/°C	Molecular Weight	Content
KH550	C_9_H_23_NO_3_Si	217	0.946	104.7	221.37	≥97
KH570	C_10_H_20_O_5_Si	255	1.055	88	248	≥97
KH590	C_8_H_22_N_2_O_2_Si	256	0.965	93	206.36	≥95

**Table 8:** Separation test results of modified asphalt binder under different storage times.

	Time Asphalt Samples	*B*	*B_n-_*_570-0.2_	*B_n-_*_570-0.3_	*B_n-_*_570-0.4_	*B_n-_*_570-0.5_
Softening point difference (°C)	3 h	1.6	1.3	1.2	1.1	1.1
	6 h	4.6	3.7	2.4	1.9	1.8
	12 h	5.6	4.5	3.3	2.4	2.2
	24 h	5.8	4.6	3.3	2.5	2.2

**Table 9:** Fatigue life test results and fatigue life model of modified bagasse fiber asphalt mixtures at different strain levels.

Solution	Sample	*N_f-_*_450_	*N_f-_*_750_	*N_f-_*_1050_	Fatigue Model	R^2^
Water	*B*	10,671	2830	1488	4.5 × 10^10^ (ε)^−2.496^	0.9993
	*B_n-_*_570-0.2_	14,030	3449	2784	2.85 × 10^10^ (ε)^−2.738^	0.9999
	*B_n-_*_570-0.3_	36,942	8030	3169	2.67 × 10^12^ (ε)^−2.962^	0.9998
	*B_n-_*_570-0.4_	47,299	10,559	3519	3.60 × 10^12^ (ε)^−2.970^	0.9986
	*B_n-_*_570-0.5_	54,971	23,112	3841	2.39 × 10^10^ (ε)^−2.125^	0.9284
NaCl	*B*	9028	2445	1272	3.14 × 10^10^ (ε)^−2.465^	0.9978
	*B_n-_*_570-0.2_	11,996	2904	2363	2.80 × 10^10^ (ε)^−2.401^	0.9657
	*B_n-_*_570-0.3_	32,077	7002	2678	2.37 × 10^12^ (ε)^−2.966^	0.9997
	*B_n-_*_570-0.4_	41,435	9186	3144	3.23 × 10^12^ (ε)^−2.974^	0.9998
	*B_n-_*_570-0.5_	46,973	19,899	3380	1.87 × 10^10^ (ε)^−2.109^	0.9637
Na_2_SO_4_	*B*	8576	2330	1196	3.01 × 10^10^ (ε)^−2.467^	0.9991
	*B_n-_*_570-0.2_	11,280	2748	2221	2.52 × 10^10^ (ε)^−2.394^	0.9665
	*B_n-_*_570-0.3_	30,184	6665	2501	2.05 × 10^12^ (ε)^−3.012^	1
	*B_n-_*_570-0.4_	38,907	8686	2975	2.81 × 10^12^ (ε)^−3.04^	0.9998
	*B_n-_*_570-0.5_	45,094	18,910	3220	1.96 × 10^10^ (ε)^−2.709^	0.9303

Note: *N_f-_*_450_, *N_f-_*_750_, and *N_f-_*_1000_ represent the fatigue lives at strain levels of 450, 750, and 1000, respectively.

**Table 10:** Test results of modified bagasse fiber asphalt and aggregate surface free energy at 0.4% doping.

Erosion Environment	Surface Tension	*W_Spread_*	*W_Er-strip_*	*W_Co-strip_*
Water	72.80	79.00	−2.45	76.55
10%NaCl	75.93	78.84	−3.51	75.33
10%Na_2_SO_4_	76.12	81.95	−8.30	73.65

## Data Availability

The data presented in this study are available on request from the corresponding author.
